# Inception Modules Enhance Brain Tumor Segmentation

**DOI:** 10.3389/fncom.2019.00044

**Published:** 2019-07-12

**Authors:** Daniel E. Cahall, Ghulam Rasool, Nidhal C. Bouaynaya, Hassan M. Fathallah-Shaykh

**Affiliations:** ^1^Department of Electrical and Computer Engineering, Rowan University, Glassboro, NJ, United States; ^2^Departments of Neurology and Mathematics, University of Alabama at Birmingham, Birmingham, AL, United States

**Keywords:** gliomas, brain tumor segmentation, fully convolutional neural network, inception, U-net

## Abstract

Magnetic resonance images of brain tumors are routinely used in neuro-oncology clinics for diagnosis, treatment planning, and post-treatment tumor surveillance. Currently, physicians spend considerable time manually delineating different structures of the brain. Spatial and structural variations, as well as intensity inhomogeneity across images, make the problem of computer-assisted segmentation very challenging. We propose a new image segmentation framework for tumor delineation that benefits from two state-of-the-art machine learning architectures in computer vision, i.e., Inception modules and U-Net image segmentation architecture. Furthermore, our framework includes two learning regimes, i.e., learning to segment intra-tumoral structures (necrotic and non-enhancing tumor core, peritumoral edema, and enhancing tumor) or learning to segment glioma sub-regions (whole tumor, tumor core, and enhancing tumor). These learning regimes are incorporated into a newly proposed loss function which is based on the Dice similarity coefficient (DSC). In our experiments, we quantified the impact of introducing the Inception modules in the U-Net architecture, as well as, changing the objective function for the learning algorithm from segmenting the intra-tumoral structures to glioma sub-regions. We found that incorporating Inception modules significantly improved the segmentation performance (*p* < 0.001) for all glioma sub-regions. Moreover, in architectures with Inception modules, the models trained with the learning objective of segmenting the intra-tumoral structures outperformed the models trained with the objective of segmenting the glioma sub-regions for the whole tumor (*p* < 0.001). The improved performance is linked to multiscale features extracted by newly introduced Inception module and the modified loss function based on the DSC.

## 1. Introduction

In recent years, there has been a proliferation of machine and especially deep learning techniques in the medical imaging field (Litjens et al., 2017). Deep learning algorithms also referred to as deep neural networks, are built using large stacks of individual artificial neurons, each of which performs primitive mathematical operations of multiplication, summation, and thresholding. One of the key reasons for the success of these modern deep neural networks is the idea of representation learning; the process of learning useful features automatically from the data as opposite to manual selection by expert humans (LeCun et al., 2015). Specifically, a convolutional neural network (CNN) is designed to extract features from two-dimensional grid data, e.g., images, through a series of learned filters and non-linear activation functions. The set of features learned through this process can then be used to perform various downstream tasks such as image classification, object detection, and semantic or instance segmentation (LeCun et al., 2015).

Recently, U-Net (Ronneberger et al., 2015) which is an end-to-end fully convolutional network (FCN) (Long et al., 2015) was proposed for semantic segmentation of various structures in medical images. U-Net architecture is built using a contracting path, which captures high-resolution, contextual features while downsampling at each layer, and an expanding path, which increases the resolution of the output through upsampling at each layer (Ronneberger et al., 2015). The features from the contracting path are combined with features from the expanding path through skip connections (Drozdzal et al., 2016), ensuring localization of the extracted contextual features. Originally the U-Net was developed and applied to cell tracking, more recently the model has been applied to other medical segmentation tasks, such as, brain vessel segmentation (Livne et al., 2019), brain tumor segmentation (Dong et al., 2017), and retinal segmentation (Girard et al., 2019). Architectural variations and extensions of the U-Net algorithm, such as 3D U-Net (Kamnitsas et al., 2017; Sandur et al., 2018), H-DenseUNet (Li et al., 2018), RIC-UNet (Zeng et al., 2019), and Bayesian U-Net (Orlando et al., 2019) have been developed to tackle different segmentation problems in the medical imaging community.

Accurate semantic segmentation depends on the extraction of local structural as well as global contextual information from medical images during the learning process (training). Therefore, various multi-path architectures have been proposed in the medical image segmentation literature which extract information from given data at multiple scales (Havaei et al., 2017; Kamnitsas et al., 2017; Salehi et al., 2017). The concept of extracting and aggregating features at various scales has also been accomplished by Inception modules (Szegedy et al., 2015). However, the mechanism of feature extraction is different compared to multi-path architectures (Havaei et al., 2017; Kamnitsas et al., 2017; Salehi et al., 2017). Each Inception module applies filters of various sizes at each layer and concatenates resulting feature maps (Szegedy et al., 2015). The dilated residual Inception (DRI) block introduced in Shankaranarayana et al. (2019) was designed to accomplish multi-scale feature extraction in an end-to-end, fully convolutional retinal depth estimation model. The MultiResUNet recently proposed in Ibtehaz and Rahman (2019) combined a U-Net with residual Inception modules for multi-scale feature extraction; authors applied their architecture to several multimodal medical imaging datasets. Integrating Inception modules in a U-Net architecture has also been evaluated in the context of left atrial segmentation (Wang et al., 2019). An architecture proposed in Li and Tso (2018) for liver and tumor segmentation also incorporated inception modules, along with dilated Inception modules, in a U-Net. Concurrently and independently of this work, inception modules within U-Net have also been recently proposed for brain tumor segmentation in Li et al. (2019). However, authors used a cascade approach, i.e., first learn the whole tumor, then learn the tumor core, and finally learn the enhancing tumor, which requires three different models. Our proposed architecture is an end-to-end implementation with respect to all tumor subtypes.

The Multimodal Brain Tumor Image Segmentation (BRATS) challenge, started in 2012, has enabled practitioners and machine learning experts to develop and evaluate approaches on a continuously growing multi-class brain tumor segmentation benchmark (Menze et al., 2014). Based on the annotation protocol, deep learning architectures designed for the problem typically derive the segmentation using a pixel-wise softmax function on the output feature map (Isensee et al., 2018a). The softmax function enforces mutual exclusivity, i.e., a pixel can only belong to one of the intra-tumoral structures. The individual output segments are then combined to create the glioma sub-regions. Learning the glioma sub-regions directly using a pixel-wise sigmoid function on the output feature map has been discussed in Isensee et al. (2018b), as well as in Wang et al. (2018) using a cascaded approach.

In this work, we introduce an end-to-end brain tumor segmentation framework which utilizes a modified U-Net architecture with Inception modules to accomplish multi-scale feature extraction. Moreover, we evaluate the impact of training various models to segment the glioma sub-regions directly rather than the intra-tumoral structures. Both learning regimes were incorporated into a new loss function based on the Dice similarity Coefficient (DSC).

## 2. Methods

### 2.1. Data and Preprocessing

All experiments were conducted on the BRATS 2018 dataset (Menze et al., 2014; Bakas et al., 2017a,b,c, 2018), which consists of magnetic resonance imaging (MRI) data of 210 high-grade glioma (HGG) and 75 low-grade glioma (LGG) patients. Each patient's MRI data contained four MRI sequences: T2-weighted (T2), T1, T1 with gadolinium enhancing contrast (T1C), and Fluid-Attenuated Inversion Recovery (FLAIR) images. Furthermore, pixel-level manual segmentation markings are provided in the BRATS dataset for three *intra-tumoral* structures: necrotic and non-enhancing tumor core (label = 1), peritumoral edema (label = 2), and enhancing tumor (label = 4). For the intra-tumoral structures, following *glioma sub-regions* (Menze et al., 2014) were defined: whole tumor (WT) which encompasses all three intra-tumoral structures (i.e., label = 1∪2∪4), tumor core (TC) that contains all but the peritumoral edema (i.e., label = 1∪4), and enhancing tumor (ET) (label = 4). Different sequences provide complementary information for identifying the intra-tumoral structures: FLAIR highlights the peritumoral edema, T1C distinguishes the ET, and T2 highlights the necrotic and non-enhancing tumor core. Converting from the intra-tumoral structures to the glioma sub-regions is a linear, reversible transformation; the glioma sub-regions are generated from the intra-tumoral structures, and provided the glioma sub-regions, the original intra-tumoral structures can be recovered.

The BRATS dataset is provided in a preprocessed format, i.e., all the images are skull-stripped, resampled to an isotropic 1 mm^3^ resolution, and all four modalities of each patient are co-registered. We performed additional preprocessing that included (in order): (1) obtaining the bounding box of the brain in each image, and extracting the selected portion of the image, effectively zooming in on the brain and disregarding excess background pixels, (2) re-sizing the cropped image to 128 x 128 pixels, (3) removing images which contained no tumor regions in the ground truth segmentation, (4) applying an intensity windowing function to each image such that the lowest 1% and highest 99% pixels were mapped to 0 and 255, respectively, and (5) normalizing all images by subtracting the mean and dividing by the standard deviation of the dataset.

### 2.2. Segmentation Model Architecture

**Figure 1:**
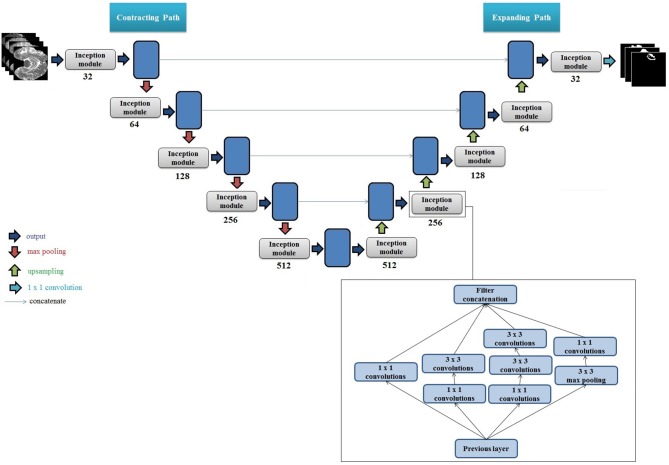
Cartoon of the proposed segmentation architecture. The set of numbers shown below each Inception module indicate total number of filters used, and height, width, and depth of the input feature map. The number of filters at each layer double on the encoder side, and the size of the output feature map (height and width) halve. The multiplication by 4 for each depth value is due to the 4 filter variations in the Inception module, which generates 4 sets of equally sized feature maps that are concatenated. The feature maps are then downsampled using max pooling, which halves their height and width. This process is repeated until reaching the bottleneck i.e., the "center" of the U. Upsampling is then performed which doubles the height and width of each feature map, and the feature maps from the corresponding stage on the contracting path are concatenated to the upsampled feature maps (shown by blue lines). The concatenation of the feature maps from the contracting path doubles the depth of the output feature map on the expanding path, hence the multiplication by 8. At the last layer on the expanding path, the output height and width are equivalent to the height and width of the original input images. A set of 1 × 1 convolutions is then applied to reduce the depth of the last feature map to equal the number of classes (tumor regions). A pixel-wise activation function is then applied to then convert the reduced feature map to binary segmentation images. Right Bottom: Internal architecture of one Inception module with multiple convolutional filters and max pooling filters is presented. The numbers in each block represent convolution filter size. We used two 3 × 3 filters in series to get an equivalent receptive field of a 5 × 5 convolutional filter.

We propose a new architecture based on the 2D U-Net and factorized convolution Inception module (Ronneberger et al., 2015; Szegedy et al., 2016). Each convolutional layer in the original U-Net was replaced with an Inception module that included multiple sets of 3 × 3 convolutions, 1 × 1 convolutions, 3 × 3 max pooling, and cascaded 3 × 3 convolutions. A cartoon of the proposed network architecture with an expanded view of the Inception module is presented in Figure 1. We note that at each layer on the contracting path, the height and width of the feature maps are halved and the depth is doubled until reaching the bottleneck i.e., the center of the "U." Conversely, on the expanding path, the height and width of the feature maps are doubled and the depth is halved at each layer until reaching the output (i.e., segmentation mask for the given input image). Furthermore, each set of feature maps generated on the contracting path are concatenated to the corresponding feature maps on the expanding path. We used rectified linear unit (ReLU) as the activation function for each layer, and performed batch normalization (Ioffe and Szegedy, 2015) in each Inception module.

The input to our model is an *N* × *M* × *D* pixel image and the output of the model is an *N* × *M* × *K* tensor. In out settings, *N* = *M* = 128 pixels, *D* = 4 which represents all four MRI modalities, and *K* = 3 which represents total number of segmentation classes, i.e., intra-tumoral structures or the glioma sub-regions. Each slice of *K* is a binary image representing the predicted segments for the ith class where 0 ≤ *i* ≤ *K*−1. The binary images are generated by pixel-wise activation functions, i.e., sigmoid for glioma sub-regions and softmax for intra-tumoral structures.

### 2.3. Evaluation Metric and Objective (Loss) Function

Dice Similarity Coefficient (DSC) is extensively used for the evaluation of segmentation algorithms in medical imaging applications (Bakas et al., 2017a). The DSC between a predicted binary image *P* and a ground truth binary image *G*, both of size *N* × *M* is given by:

(1) $$\begin{array}{r} DSC\left(P,G\right)=2\frac{\sum_{i=0}^{N-1}\sum_{j=0}^{M-1}P_{ij}G_{ij}}{\sum_{i=0}^{N-1}\sum_{j=0}^{M-1}P_{ij}+\sum_{i=0}^{N-1}\sum_{j=0}^{M-1}G_{ij}}, \end{array}$$

where *i* and *j* represent pixel indices for the height *N* and width *M*. The range of DSC is [0, 1], and a higher value of DSC corresponds to a better match between the predicted image *P* and the ground truth image *G*.

Our objective function (or the loss function) for the proposed learning algorithm consisted of a modified version of DSC (Equation 1). Specifically, following modification were made: (1) we changed the sign of the DSC coefficient to formulate a standard deep learning optimization (minimization) problem, (2) introduced log function, and (3) introduced a new parameter γ to cater for extremely large values of the loss function. For example, if a ground truth segment had very few white pixels $\overset{N-1}{\underset{i=0}{\sum }}\overset{M-1}{\underset{j=0}{\sum }}G_{ij}\approx 0$, the model may predict no white pixels $\overset{N-1}{\underset{i=0}{\sum }}\overset{M-1}{\underset{j=0}{\sum }}P_{ij}=0$ resulting in an extremely large loss function. In our preliminary experiments, we found empirically that γ = 100 provided the best segmentation performance. The resulting expression for the loss function is given as:

(2) $$\begin{array}{r} L_{DSC}\left(P,G\right)=-\log\left[2\frac{\sum_{i=0}^{N-1}\sum_{j=0}^{M-1}P_{ij}G_{ij}+\gamma }{\sum_{i=0}^{N-1}\sum_{j=0}^{M-1}P_{ij}+\sum_{i=0}^{N-1}\sum_{j=0}^{M-1}G_{ij}+\gamma }\right]. \end{array}$$

The loss function presented in Equation (2) is able to handle binary cases only (e.g., tumor and not tumor). The same can be extended for the multi-class cases as:

(3) $$\begin{array}{r} L_{DSC}\left(P,G\right)=-\log\left[\frac{1}{K}\overset{K-1}{\underset{i=0}{\sum }}DSC\left(P_{i},G_{i}\right)\right], \end{array}$$

where *K* is the total number of classes.

### 2.4. Experimental Setup and Model Training

We performed an ablation study to quantify the effects of introducing Inception modules in the U-Net architecture as well as the impact of different segmentation objectives, i.e., learning to segment intra-tumoral structures or glioma sub-regions. Specifically, we trained four different models, i.e., two variations of the U-Net architecture (with intra-tumoral structures and glioma sub-regions) and two variations of the U-Net with Inception module (intra-tumoral structures and glioma sub-regions).

We trained all four models under same conditions to ensure consistency and a fair comparison. All four models were trained using *k*-fold cross-validation. The dataset was randomly split into *k* mutually exclusive subsets of equal or near equal size. Each algorithm was run *k* times subsequently, each time taking one of the *k* splits as the validation set and the rest as the training set. In our experiments, we set *k* = 10, which means that each model was trained 10 times using a different set of 90% of the data and validated on the remaining 10% data. In total, our experimental setup generated 40 models, i.e., 10 variations per model. Later, mean and standard deviation (SD) were calculated and are reported for each model in the Results section.

We used stochastic gradient descent with an adaptive moment estimator (Adam) for training all models and their variations (Kingma and Ba, 2014). The initial learning rate was set to 10^−4^ which was exponentially decayed every 10 epochs. The batch size was set to 64 and each model was trained for 100 epochs. All learnable parameters, i.e., weights and biases of the models were initialized based on the He initialization method (He et al., 2015). The Keras (Chollet et al., 2015) application programming interface (API) with TensorFlow (Abadi et al., 2016) backend was used for implementation of all models. All models were trained on a Google Cloud Compute instance with 4 NVIDIA TESLA P100 graphical processing units (GPUs).

### 2.5. Model Testing and Statistical Analysis of Results

After training, each model was tested on the entire BRATS 2018 dataset. For the models which learned to segment the intra-tumoral structures, the predicted intra-tumoral structure segments were combined to produce the glioma sub-regions, and DSC for each glioma sub-region was computed. For models which learned to segment the glioma sub-regions directly, DSC values were readily computed. The process was repeated for each image, and after evaluating all images, the average DSC score was calculated for each glioma sub-region. Overall, the process resulted in 4 sets of 10 DSC scores, one for each glioma sub-region. All four models were compared for statistical significance using a two-tailed Student's *t*-test with equal variance and with the probability of Type-I error set to α = 0.05.

## 3. Results

**Figure 2:**
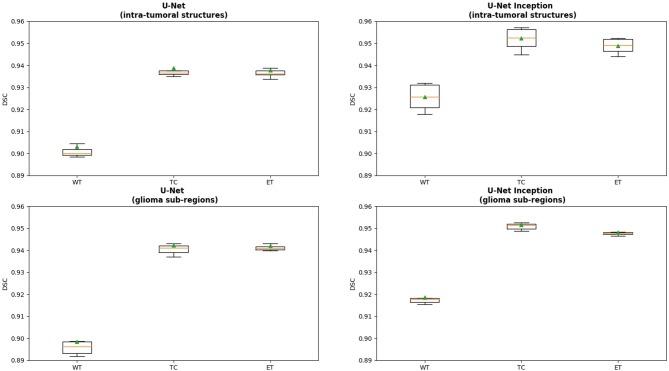
Box plot displaying the results for each model variation. The x-axis is the glioma sub-region, and the y-axis is the DSC. The median value is denoted by the horizontal orange line, and the mean is denoted by the green triangle. Abbreviations used are: WT, Whole Tumor; TC, Tumor Core; and ET, Enhancing Tumor.

We present cross-validation DSC for all four models that were trained and tested on the BRATS 2018 dataset. In Figure 2, we provide a box plot for each model variation. The glioma sub-region is on the x-axis and the DSC is on the y-axis for each plot. We note that for intra-tumoral structures, adding Inception modules to the U-Net resulted in statistically significant improvements in WT (DSC improved from 0.903 to 0.925, *p* < 0.001), TC (0.938 to 0.952, *p* < 0.001), and ET (0.937 to 0.948, *p* < 0.001). Similarly, for the glioma sub-regions, adding Inception modules to the U-Net also resulted in statistically significant improvements in WT (0.898 to 0.918, *p* < 0.001), TC (0.942 to 0.951, *p* = 0.001), and ET (0.942 to 0.948, *p* = 0.002).

**Figure 3:**
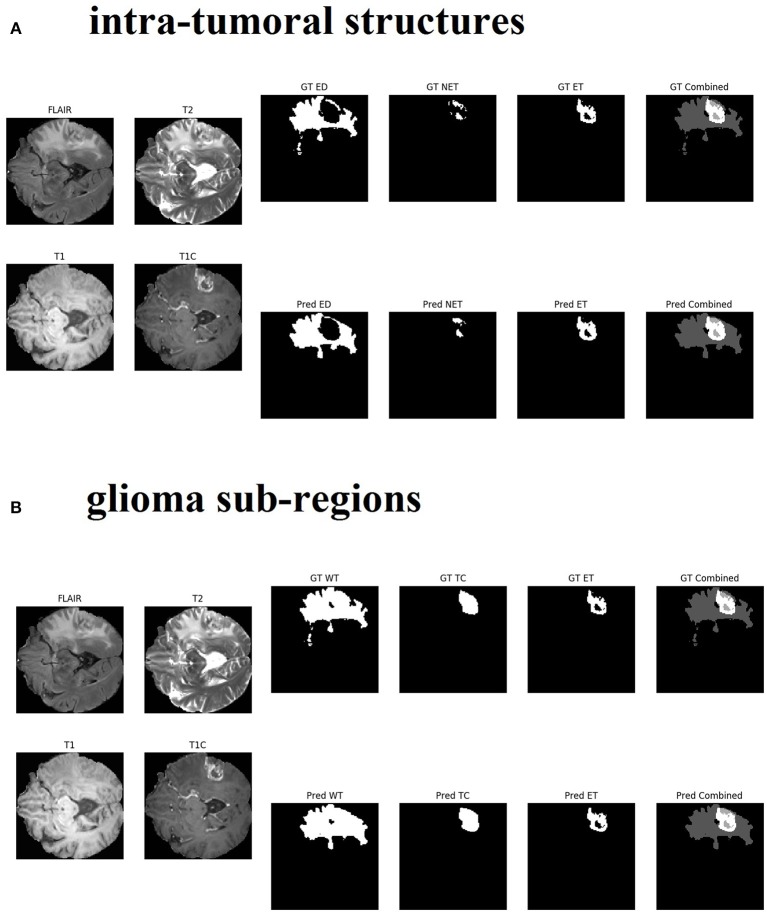
Qualitative results from the same patient are presented in sub-figure **(A)** (top, intra-tumoral structures) and **(B)** (bottom, glioma sub-regions). All four MR modalities (FLAIR, T2, T1, and T1C) are shown on the left in both sub-figures for easy visual analysis. **(A)** On the right top row, the ground truth (GT) segments for each intra-tumoral structure are presented (abbreviations used are: ED, peritumoral edema; NET, necrotic and non-enhancing tumor core; ET, enhancing tumor). On the right bottom row, the predicted (Pred) segments for each intra-tumoral structure are shown. The last image in each row is the combined segments i.e., ED, NET, and ET all in one image, distinguished by different gray-level pixel values. **(B)** On the right top row, the ground truth (GT) segments for each glioma sub-region are presented (abbreviations used are: WT, whole tumor; TC, tumor core; ET, enhancing tumor). On the right bottom row, the predicted (Pred) segments for each glioma sub-region are shown. The last image in each row is the combined segments i.e., WT, TC, and ET all in one image, distinguished by different gray-level pixel values.

Changing the objective from learning the intra-tumoral structures to learning the glioma sub-regions in the U-Net resulted in no difference in performance for WT (0.903 to 0.898, *p* = 0.307), TC (0.938 to 0.942, *p* = 0.284), and ET (0.937 to 0.942, *p* = 0.098). However, U-Net with Inception modules which learned the intra-tumoral structures outperformed U-Net with Inception modules which learned the glioma sub-regions in WT (0.918 to 0.925, *p* = 0.007), but there was no performance difference for TC (0.952 to 0.951, *p* = 0.597) and ET (0.948 to 0.948, *p* = 0.402). Qualitative results on the same patient from a U-Net with Inception modules which learned the intra-tumoral structures and U-Net with Inception modules which learned the glioma sub-regions are presented in Figures 3A,B, respectively. In Table 1, we provide a summary of statistical comparisons, i.e., *p*-values from Student's *t*-test performed to compare different models. Statistically significant *p*-values are in shown bold font.

**Table 1:** Results of statistical comparison, i.e., *p*-values from two-tailed t-tests comparing the models in the first column with the models in the second columns.

**Model 1**	**Model 2**	***p*****-values**		
		**WT**	**TC**	**ET**
U-Net intra-tumoral structures	U-Net glioma sub-regions	0.307	0.284	0.098
	U-Net Inception intra-tumoral structures	<**0.001**	<**0.001**	<**0.001**
U-Net Inception glioma sub-regions	U-Net glioma sub-regions	<**0.001**	**0.001**	**0.002**
	U-Net Inception intra-tumoral structures	**0.007**	0.597	0.402

*Statistically significant p-values are present in bold font*.

## 4. Discussion and Conclusions

We set out to tackle the challenging problem of pixel-level segmentation of brain tumors using MRI data and deep learning models. We introduced a new framework building on well-known U-Net architecture and Inception modules. We explored two different learning objectives: (1) learning to segment glioma sub-regions (WT, TC, and ET), and (2) learning to segment intra-tumoral structures (necrotic and non-enhancing tumor core, peritumoral edema, and enhancing tumor). Both learning objectives were incorporated into the newly proposed DSC based loss function. Our framework resulted into four different model variations, i.e., (1) a U-Net with learning objective of intra-tumoral structures, (2) U-Net with glioma sub-regions, (3) U-Net with Inception module and intra-tumoral structures, and finally (4) U-Net with Inception module and learning objective of glioma sub-regions.

We found that integrating Inception modules in the U-Net architecture resulted in statistically significant improvement in tumor segmentation performance that was quantified using *k*-fold cross-validation (*p* < 0.05 for all three glioma sub-regions). We consider that the observed improvement in the validation accuracy is linked to multiple convolutional filters of different sizes employed in each Inception module. These filters are able to capture and retain contextual information at multiple scales during the learning process, both in the contracting as well as expanding paths. We also consider that the improvement in the tumor segmentation accuracy is linked to the new loss function based on the modified DSC (i.e., Equation 3). In our proposed framework, we evaluate our models using DSC and the learning objective or the loss function (Equation 3) used for training these algorithms is also based on DSC. This is in contrast with conventional deep learning paradigms being used in natural image segmentation, such as, Mask R-CNN, where the loss function is based on multi-class cross-entropy and the evaluation metric is based on Intersection-over-Union (IoU) or DSC score (He et al., 2017). Furthermore, our DSC scores for each glioma sub-region on the BRATS 2018 training dataset are comparable or exceed the results of other recent published architectures such as the No New-Net, which achieved second place in the BRATS 2018 competition (Isensee et al., 2018b), and the ensemble approach proposed in Kao et al. (2018).

Our results also demonstrate that changing the learning objective from intra-tumoral structures to glioma sub-regions in the architectures with Inception modules produced a statistically significant positive impact only on WT, while not affecting TC and ET. Since the only difference between TC and WT is the peritumoral edema, these results suggest that learning to segment the peritumoral edema independently is more effective than learning in context of other two intra-tumoral structures. We hypothesize that learning to segment WT directly may be difficult for the model because it requires extracting information from multiple modalities (T1, T1C, T2, and FLAIR); however, the segmentation of peritumoral edema alone can primarily be learned from FLAIR data. Therefore, for the proposed framework, we recommend using intra-tumoral structures for learning with U-Net Inception architecture.

## Data Availability

The BRATS 2018 training dataset analyzed for this study can be found in the Image Processing Portal of the CBICA@UPenn [https://ipp.cbica.upenn.edu/].

## Author Contributions

The architecture was conceived by DC. The experiments were designed by GR, NB, and HF-S. The data was analyzed by HF-S and DC conducted the experiments and wrote the manuscript with support from GR, NB, and HF-S. All authors provided critical feedback and helped shape the research, analysis, and manuscript.

### Conflict of Interest Statement

The authors declare that the research was conducted in the absence of any commercial or financial relationships that could be construed as a potential conflict of interest.
